# Glide-Symmetric Holey Structures Applied to Waveguide Technology: Design Considerations †

**DOI:** 10.3390/s20236871

**Published:** 2020-12-01

**Authors:** Zvonimir Sipus, Katarina Cavar, Marko Bosiljevac, Eva Rajo-Iglesias

**Affiliations:** 1 Faculty of Electrical Engineering and Computing, University of Zagreb, Unska 3, 10000 Zagreb, Croatia; katarinacavar2@gmail.com (K.C.); marko.bosiljevac@fer.hr (M.B.); 2 Signal Theory and Communications Department, University Carlos III of Madrid, 28911 Leganés, Spain; eva@tsc.uc3m.es

**Keywords:** higher symmetries, glide symmetry, periodic structures, mode matching, dispersion analysis

## Abstract

Recently, there has been an increased interest in exploring periodic structures with higher symmetry due to various possibilities of utilizing them in novel electromagnetic applications. The aim of this paper is to discuss design issues related to the implementation of holey glide-symmetric periodic structures in waveguide-based components. In particular, one can implement periodic structures with glide symmetry in one or two directions, which we differentiate as 1D and 2D glide symmetry, respectively. The key differences in the dispersion and bandgap properties of these two realizations are presented and design guidelines are indicated, with special care devoted to practical issues. Focusing on the design of gap waveguide-based components, we demonstrate using simulated and measured results that in practice it is often sufficient to use 1D glide symmetry, which is also simpler to mechanically realize, and if larger attenuation of lateral waves is needed, a diagonally directed 2D glide symmetric structure should be implemented. Finally, an analysis of realistic holes with conical endings is performed using a developed effective hole depth method, which combined with the presented analysis and results can serve as a valuable tool in the process of designing novel electrically-large waveguide-based components.

## 1. Introduction

Recent research related to new electromagnetic structures and manufacturing technologies has inspired numerous new developments in the field of periodic structures due to the possibility of realizing components with properties that cannot be obtained using classical materials. Higher symmetries, i.e., periodic structures that are invariant after a translation and a second geometrical operation [1,2,3], represent a class of periodic structures with additional possibilities in creating new designs, for example in art (medieval Moorish tessellations in the Alhambra, Spain, or the graphic work of M.C. Echer), in numerical geometry (space tessellation and meshing), and in electromagnetics, where their usage has resulted in enhancing the performance of electromagnetic devices [4].

Implementation of higher symmetries in an electromagnetic structure allows various manipulations of the corresponding dispersion diagram. Depending on the desired application and the way in which the periodic structure is used, there are two basic design directions. The first direction is related to the expansion of the non-dispersive part of the dispersion diagram, which is interesting for structures used for guiding waves in desired directions. The second approach is applied when we wish to extend the bandgap in the realized dispersion diagram, for example in structures based on preventing the wave propagation in some directions. Possible applications are numerous—from lenses (e.g., ultra-wideband Luneburg lenses [5,6]), antennas (e.g., leaky-wave antennas with low frequency dependency [7,8] or with high-scanning range [9]), cost-efficient gap waveguide technology [10], phase shifters [11,12,13], filters [13,14], or contactless flanges in the mm-wave frequency range with low leakage [15].

Although there has been intensive research on periodic structures with higher symmetries over the last five years, there are still many theoretical and practical questions that need to be discussed. Extensive theoretical modeling based on the mode matching approach has shown the advantages and limitations of these structures, and this is in agreement with the results obtained using general numerical solvers [10,16,17,18,19,20,21]. The focus of these works was on geometries that have glide symmetry in two directions. However, it is possible to have glide symmetry in only one direction, resulting in simpler structures. Thus, it is of interest to analyze how this “simple” approach of implementing glide symmetry (i.e., reduced glide symmetry), which is easier to implement in practice, compares to a full glide symmetry. Furthermore, the base geometrical elements for the realization of these periodic structures are usually cylindrical or rectangular holes. Parametric analyses of the influence that the geometry of these holes has on the dispersion diagram have been extensively reported [16,22,23,24]; however, in practical realization, drilling of the holes usually means that the actual geometry of the hole is not a perfect cylinder, but rather a cylinder with a conical ending. For this reason we are interested in to what extent the conical ending of the holes influences the characteristics of the designed device. Therefore, the aim of this paper is to make a contribution toward these two practical topics.

The paper is organized as follows. First, holey parallel plate waveguides with implemented one-dimensional (1D) and two-dimensional (2D) glide symmetries are discussed. The focus will be on the differences in obtainable dispersion diagrams. In Section 3 we discuss practical issues related to properties of waveguide components whose design utilizes bandgap properties of holey glide-symmetric structures, and the results of the experimental prototype are presented. Finally, the discussion will focus on another practical issue related to holey waveguide components with a conical shape of the hole bottom in order to also cover this aspect in the realization of components with higher-symmetry periodic structures.

## 2. Glide-Symmetric Holey Parallel Plate Waveguide

A periodic structure possesses a higher symmetry when more than one geometrical operation is needed for the unit cell to coincide with itself. A common example is glide symmetry, which indicates the invariance of a periodic structure under a translation for half of its period and a mirroring with respect to a symmetry plane defined by the considered structure. Other kinds of higher symmetries are defined by a combination of translation and rotation (twist or screw symmetry) [25] or by time operation (parity-time symmetry) [26].

**Figure 1:**
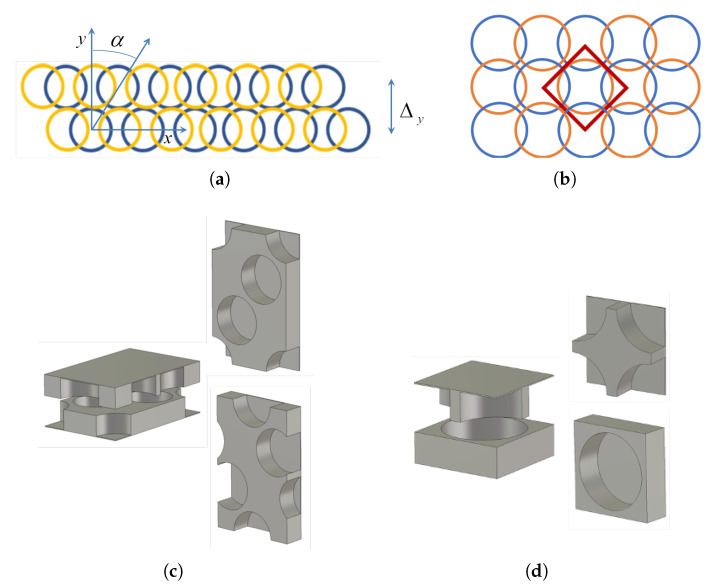
Holey glide-symmetric structures: (**a**) sketch of 1D glide symmetric structure; (**b**) sketch of 2D glide symmetric structure—yellow and blue circles denote holes at the top and bottom plates of parallel-plate waveguide (PPW); (**c**) sketch of periodic unit cell of 1D glide symmetric PPW structure; (**d**) sketch of periodic unit cell of 2D glide symmetric PPW structure.

In parallel-plate waveguide (PPW) applications, a glide symmetry operator should be applied along the waveguide, and there is a degree of freedom in defining the glide-symmetric periodic cell. Two possibilities will be considered, both of them having advantages in particular applications. To define them we introduce two versions of glide operators, 1D and 2D, which can be defined in the following way (the coordinate system is given in Figure 1):

(1) $$1D\mathrm{glide}\mathrm{operator}:\left\{\begin{array}{c} \left(x,y\right)\;⟶\;\left(x+\frac{P_{x}}{2},y\right)\\ z\;⟶\;-z \end{array}\right.$$

(2) $$2D\mathrm{glide}\mathrm{operator}:\left\{\begin{array}{c} \left(x,y\right)\;⟶\;\left(x+\frac{P_{x}}{2},y+\frac{P_{y}}{2}\right)\\ z\;⟶\;-z \end{array}\right.$$

**Figure 2:**
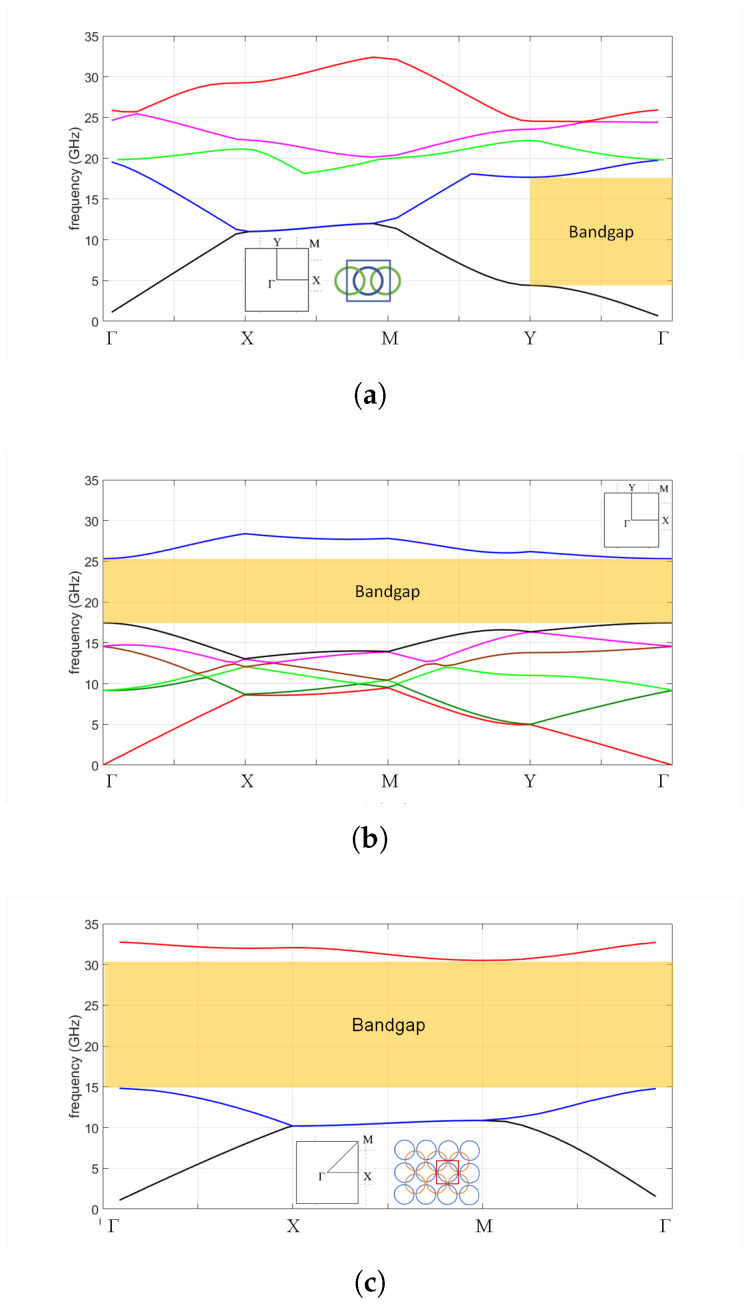
Dispersion diagram of the 1D and 2D glide symmetric structures obtained using CST Microwave Studio: (**a**) 1D case with inclination angle α = 0°; (**b**) 1D case with inclination angle α = 33.7°; (**c**) 2D glide-symmetric case.

A sketch of 1D and 2D glide operators, together with the corresponding PPW unit cells, is given in Figure 1. Note that in the 1D case we have two free parameters: the lateral distance between rows following the 1D glide symmetry $\Delta_{y}$, and the inclination angle α. Furthermore, there is no requirement for 1D structure to be periodic in the lateral direction (in the example in Figure 1b we have selected a special case, α = 33.7°, to ensure periodicity in the lateral direction, which enables calculation of the two-dimensional dispersion diagram). For an inclination angle α = 45° and $\Delta_{y}=P_{x}/2$ we get a 2D glide symmetric structure. Note that in the 2D case, the minimum unit cell is positioned in the diagonal direction (see Figure 2b). However, just to be able to compare 1D and 2D cases, we will keep the notation of periodicities in the *x*- and *y*-directions, i.e., $P^{2D}=P^{1D}/\sqrt{2}=P_{x}/\sqrt{2}$.

Differences in the unit cell geometry are also reflected in the corresponding dispersion diagrams. As an example, we considered glide symmetric periodic structures with the stop-band in the K-band. Without loss of generality we fixed the diameter and the depth of the holes (2*r* = 7 mm and *h* = 3 mm, respectively) and the gap between parallel plates (*g* = 0.1 mm). We used the period that maximizes the bandgap, and thus the $P^{2D}=9$ mm ($r/P^{2D}=0.39$) for the 2D periodic structure (see also the discussion about the optimum ratio $r/P^{2D}$ given at the end of this section). For 1D glide symmetry we considered two examples, one with zero inclination angle ( $P^{1D}=P_{x}=P^{2D}\sqrt{2}=13$ mm, α = 0°, $\Delta_{y}=P_{x}$), and one with inclination angle α = 33.7° ($\Delta_{y}=P_{x}/2$, the unit cell of this structure is shown in Figure 1c).

**Figure 3:**
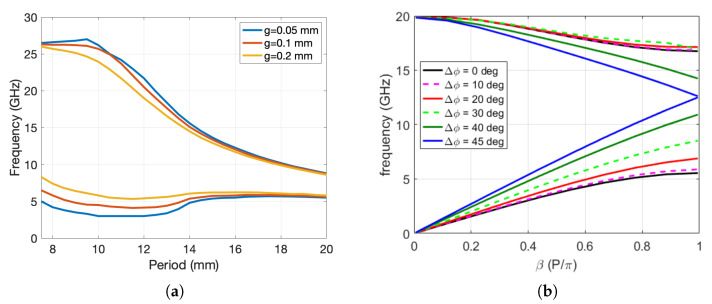
(**a**) The bandgap cut-off frequencies of a 1D glide-symmetric periodic structure (α = 0°, $\Delta_{y}=P_{x}$) as a function of periodicity $P_{x}$ and gap size *g*; (**b**) dispersion diagram of first two propagating modes of a 1D glide-symmetric periodic structure with α = 0°, $\Delta_{y}=P_{x}$, and *g* = 0.2 mm for different propagation directions of EM waves.

From Figure 2 it can be seen that the 1D holey structure for an inclination angle α = 0° acts as a soft surface, i.e., it prevents the propagation of EM waves in the direction perpendicular to the row of holes (i.e., y-direction) [27,28]. A large stop-band is obtained for the structures where the holes are large enough to overlap, i.e., in the case when there is a non-zero cross-section of the projection of the holes into the symmetry plane (i.e., 2*r* > $P^{1D}/2$). This can be seen in Figure 3a, in which the bandgap cut-off frequencies are shown as a function of periodicity $P^{1D}$ and gap size *g*. The maximum bandgap is obtained for period $P^{1D}=9.5$ mm, i.e., for $r/P^{1D}=0.37$. The stop property is present not only for waves propagating in the *y*-direction, but the bandgap is also present for EM waves propagating in the cone ±45°, as seen in Figure 3b. However, the bandgap is positioned at quite low frequencies, between 4 and 18 GHz in this case, which was not our intention in the design.

If we incline the structure in the lateral direction by an angle α, this will enable us to place the lateral walls closer. Furthermore, if the selected diameter of the holes results in mutual overlapping, i.e., if there is a non-zero cross-section of the projection into the symmetry plane of each hole in the upper plate with the projections of the four neighboring holes in the lower plate (and vice versa), then a large bandgap opens at higher frequencies in all directions, see Figure 2b. If we further increase the inclination angle to α = 45°, we will obtain the 2D glide-symmetric structure with maximum bandgap size, see Figure 2c. The dispersion diagram is now quite simple with a small number of modes, mostly because of the presence of a higher symmetry and a smaller unit cell ($P^{2D}=P^{1D}/\sqrt{2}$). One should note that implementing the periodic structure from Figure 1b and not noticing the minimum period of the structure would influence the layout of the dispersion diagram—one would obtain more propagating modes. However, the bandgap would be correctly determined.

**Figure 4:**
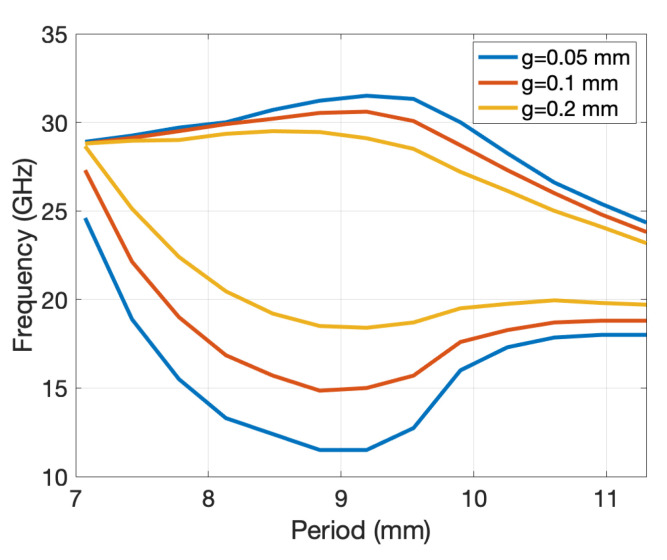
Bandgap cut-off frequencies of 2D glide symmetric structures for different periods and gap sizes; the period of the periodic structure is $P^{2D}=P_{x}/\sqrt{2}$.

The dependency of the bandgap, i.e., the dependency of the lower and upper cut-off frequency of the 2D glide-symmetric structure on the structure period and on the gap size, is given in Figure 4. It can be seen that the maximum bandgap was obtained for $P^{2D}=9$ mm, i.e., for $r/P^{2D}=0.39$, which is in agreement with results from the literature [23,29]. Furthermore, if there is no mutual overlapping of holes in the lower and upper plates (in the case of periodic structures with $P^{2D}>9.9$ mm), the bandgap is reduced.

## 3. Waveguide Components Based on Using Bandgap Properties of Holey Glide-Symmetric Structures

Undesirable EM energy leakage from certain types of waveguides, flanges, shieldings, and similar components in the mm-frequency band is often encountered and is a result of poor manufacturing, small cracks, and material wear. Such waveguide components are typically manufactured in two parts that are joined together, and ensuring very good flatness and good electric contact in these cases is mechanically a very difficult task and other solutions are investigated. Note that even typical production tolerances in surface flatness will cause small gaps when these two parts are mounted together, and in order to avoid leakage of EM energy through the gaps one needs to use a large amount of screws to ensure a firm contact (see, e.g., [30]). One alternative popular solution is based on gap-waveguide technology, which uses the electromagnetic stop-band in order to contain the EM energy inside the structure [28,31,32]. The classical gap-waveguide technology is based on manufacturing a periodic array of pins that form the stop-band in the PPW, and the benefits of using gap waveguide structures to prevent this leakage have been extensively demonstrated. Our aim is to investigate the potential of holey glide-symmetric structures in similar applications. Due to its mechanical simplicity (instead of manufacturing the pins one needs only to drill holes, which is easier and cheaper to produce) and good dispersion properties, glide-symmetric structures have great potential in these types of applications. In more detail, the waveguide components are typically fabricated in two separate parts using computer numerical control (CNC) machining or electrical discharge machining (EDM). If the CNC milling process is applied, one should note that drilling the holes is easier than milling the pins (additionally, closely-spaced thin pins are easy to break). If the EDM process is applied, the glide-symmetric holey structure is less sensitive to the fabrication tolerances due to the larger dimensions of the holes, since the periodicity of the holey structure is typically 2–3 times larger than the equivalent pin structure (which is particularly important in the higher mm-wave frequency range) [10]. Understanding these concepts and the benefits of particular realizations is crucial for the development of actual existing devices and the further development of new devices applicable in classical EM applications and also in different sensing applications.

In designing gap waveguide components based on glide-symmetric structures one would like to select dimensions that would maximize the range of the bandgap. The dependency of the lower and upper cut-off frequencies of the 2D glide-symmetric structure on the structure period and on the gap size was already discussed in Figure 4, i.e., the ratio of hole radius and structure period that maximizes the bandgap is $r/P^{2D}=0.39$ (i.e., $r/P^{1D}=0.27$). The position of the maximum does not depend on the gap size, making it easy to design components since the sizes of the gaps or cracks are not known in principle. Note that the hole depth for large enough values does not influence the bandgap range since all the modes in the circular waveguides (i.e., in the holes) are evanescent due to the subwavelength radius of the holes, and thus the EM field does not penetrate deep inside the hole.

These results indicate that glide-symmetric holey technology can be efficiently used as a simple way of solving the leakage problem due to the presence of undesired cracks and gaps in waveguide components. Additionally, it is important to highlight that it is very simple to implement holey glide-symmetric structures using the 1D approach. In more detail, from a practical point of view it is often important that a holey glide-symmetric structure occupies as little space as possible in the lateral direction due to the fact that in practical waveguide-based components we would like to put different parts close to each other and in this way to reduce the size of the considered component (a typical example is a waveguide-based feeding network of an antenna array, see, e.g., [30]). Therefore, it is advantageous not to have a half-period shift of the row of holes in the lateral direction, so even when a 2D glide-symmetric structure is implemented, it is oriented in the diagonal direction of the unit cell (i.e., following the 1D approach, see Figure 1).

**Figure 5:**
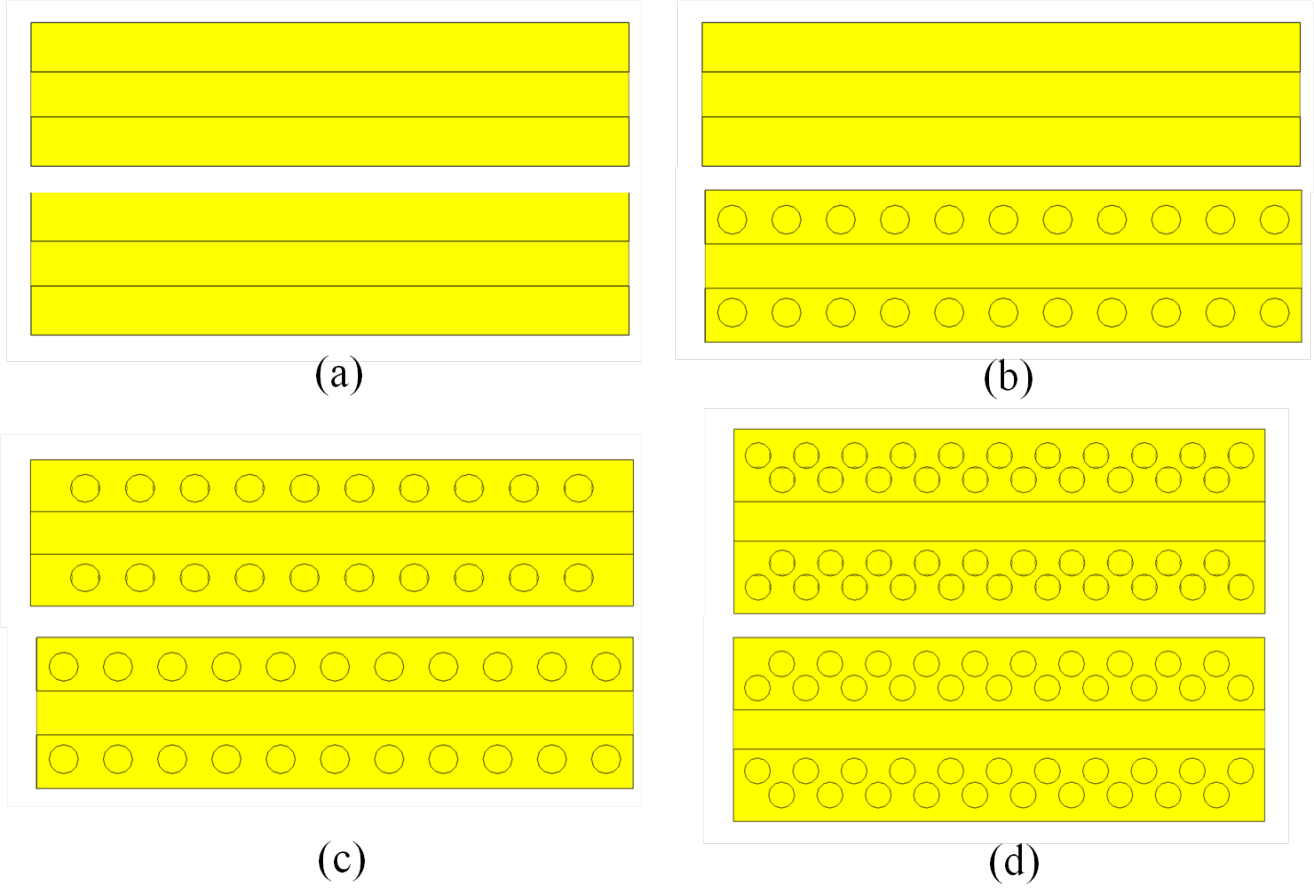
Sketch of the considered holey periodic structures: (**a**) classical waveguide with the PPW in the side walls; (**b**) waveguide with one row of holes in one plate of the PPW only; (**c**) waveguide with one row of holes in both the top and bottom plates of the PPW; (**d**) waveguide with two rows of holes in both the top and bottom plates of the PPW following the 2D glide-symmetry grid.

**Figure 6:**
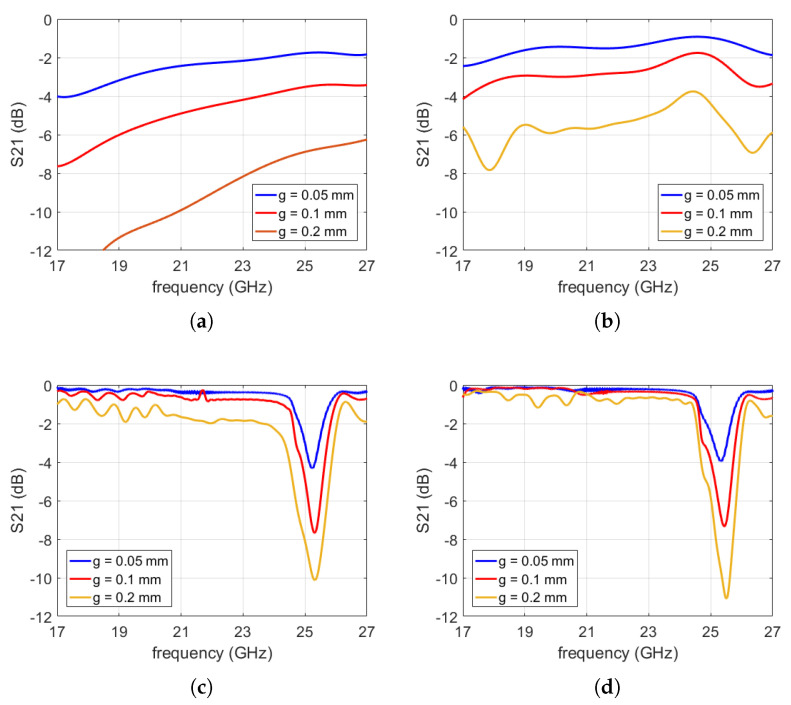
Transmission properties of a finite holey glide-symmetric waveguide (*L* = 143 mm) as a function of the gap size; the considered structures are shown in Figure 5. The period of the holey glide-symmetric structure is $P^{1D}=13$ mm; (**a**) no holes present; (**b**) structure with one row of holes in the bottom; (**c**) structure with one row of holes in the top and bottom plates (shifted half a period versus each other); (**d**) with two rows of holes in the top and bottom plates.

In order to investigate the optimum glide-symmetric topology, we considered a rectangular waveguide with a gap (i.e., with a PPW) in the lateral walls in which the holes are periodically drilled (without loss of generality the PPWs are placed in the middle of the side waveguide walls). First we need to determine the required number of rows with holes and therefore the following three holey structures were considered (see Figure 5), together with the structure without the holes as a reference case (i.e., a rectangular waveguide with two PPWs in the middle of side walls). By inspecting Figure 6, where the $S_{21}$ parameter of finite-size rectangular waveguides is shown (*L* = 143 mm), we can conclude that even a small gap in the lateral wall causes serious degradation in the transmitting waveguide properties (Figure 6a). Furthermore, it is clear that drilling holes in one plate only does not improve the transmission properties (Figure 6b). However, even one row of 1D glide symmetric holes efficiently prevents the leakage of EM energy, in particular for small sizes of the gap (Figure 6c). For larger gap sizes, two rows of glide-symmetric holes are needed (Figure 6d).

**Figure 7:**
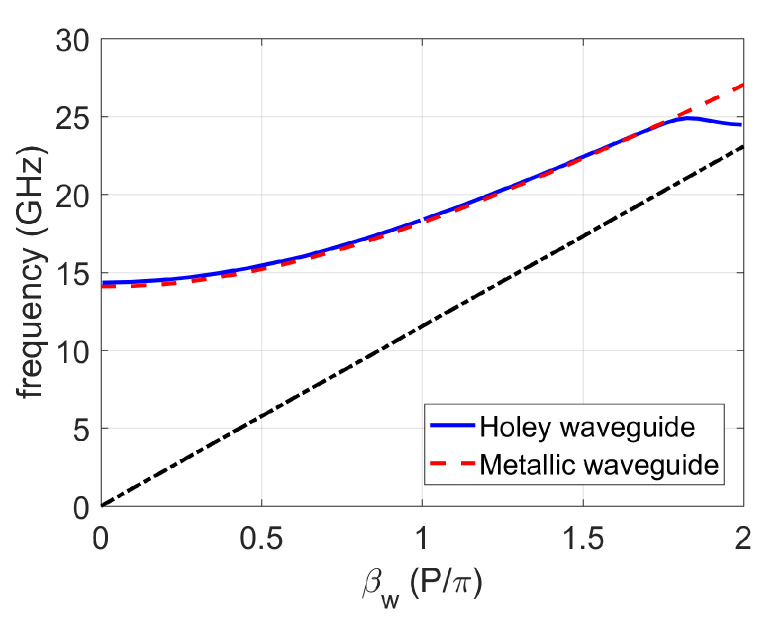
Propagation constant of the regular (fully metallic) and holey glide-symmetric waveguides. The light line is shown with a dash-dotted line.

One would expect “perfect” waveguide propagation properties inside the whole bandgap. However the analysis of a finite waveguide structure in Figure 6 shows a strong reflection above 25 GHz. The problem is in the Bragg frequency, i.e., when the period of the holes is equal to the guided wavelength of the waveguide propagating mode a strong reflection occurs since all the small reflections from the holey discontinuities are constructively added in phase. This is also visible if we plot the comparison of the propagation constant of the regular and holey glide-symmetric waveguides, see Figure 7. It can be seen that the difference is negligible until we reach the vicinity of the Bragg frequency.

**Figure 8:**
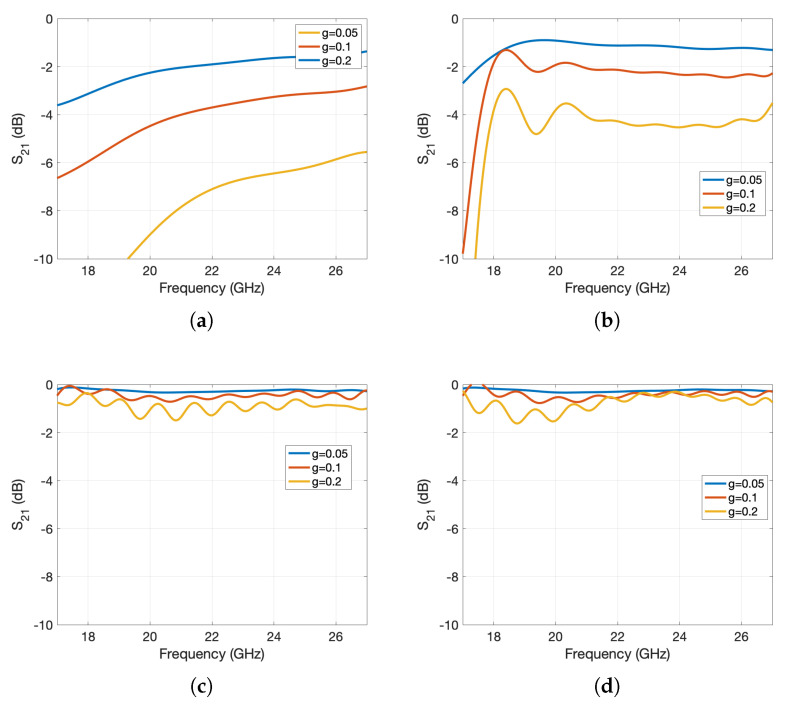
Transmission properties of a finite holey glide-symmetric waveguide (*L* = 121 mm) as a function of a gap size; the considered structures are shown in Figure 5. The period of the holey glide-symmetric structure is $P^{1D}=11$ mm; (**a**) no holes present; (**b**) structure with one row of holes in the bottom; (**c**) structure with one row of holes in the top and bottom plates (shifted half a period versus each other); (**d**) with two rows of holes in the top and bottom plates.

**Figure 9:**
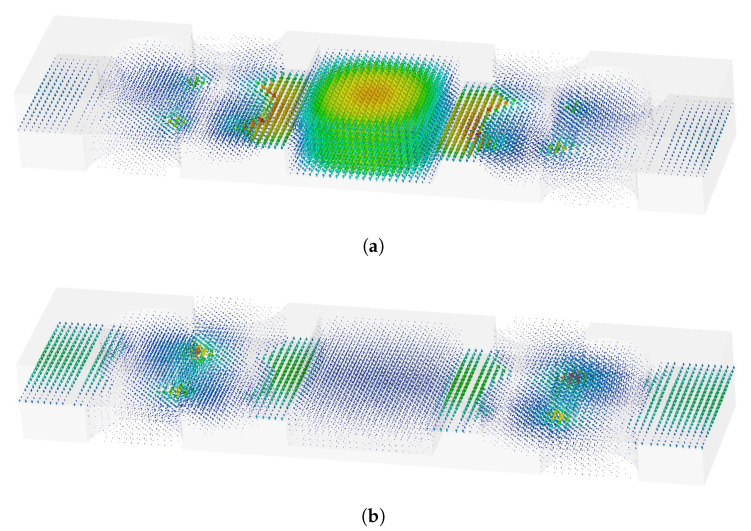
E-field distribution within the holey glide-symmetric structure of (**a**) a desired waveguide mode and (**b**) a waveguide mode that exists outside the bandgap.

The problem of Bragg frequency reflection can be mitigated by reducing the period of the holes (thus effectively moving the Bragg reflection to a higher frequency). This will reduce the bandgap, but since there is no Bragg reflection, effectively the working frequency range will be extended, as seen in Figure 8. However, the properties of the bandgap are also changed and the ripples in the transmission characteristic are visible since now more than one mode is propagating (e.g., below 22 GHz for *g* = 0.2 mm). It should be noted that this second propagating mode (due to the fact that the periodic structure is not in the bandgap) is much weaker. This is also visible in Figure 9 in which the field distribution of these two modes is given. It can be seen that the mode that exist outside the bandgap has the E-field maximum in the parallel plate region; thus it is weakly excited by the dominant waveguide mode (excitation of the whole structure). This is the case when we have a geometrically regular parallel plate structure. However, this mode will not be present in reality since the gap will not be regular, i.e., it will be present in places with imperfections of the realized waveguide component.

One can conclude that practically there is no difference in the transmission properties of structures with one and two rows of glide symmetric holes, in particular for small sizes of the gap (up to 0.1 mm in the K band). Therefore, it is enough to put one row of holes in different realizations of waveguide components in order to avoid energy leakage due to the presence of undesired cracks and gaps.

### Practical Realization of Glide-Symmetric Holey Waveguide

**Figure 10:**
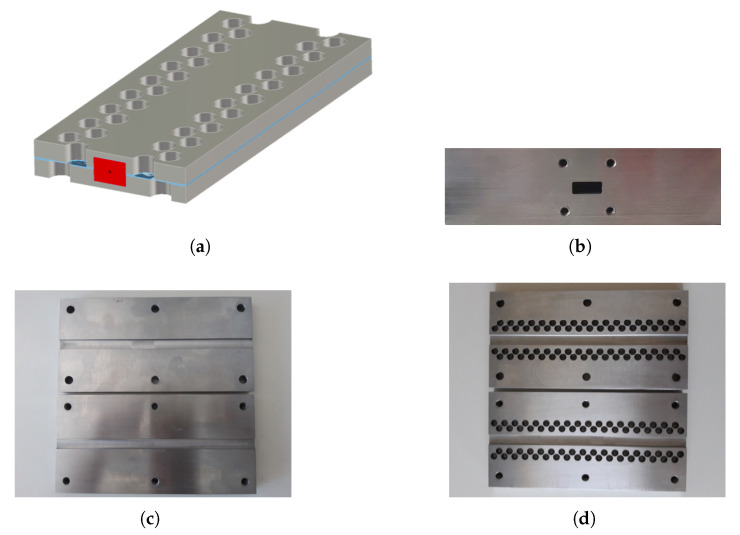
(**a**) Sketch of the developed holey glide-symmetric waveguide; (**b**) picture of the side view of the realized waveguides; (**c**) picture of the realized ordinary waveguide; (**d**) picture of the realized holey glide-symmetric waveguide.

To verify the findings from the simulated results we built two waveguide prototypes operating in the K-band. The first waveguide is a classical one, whereas the second one implements the holey gap-waveguide technology in the side walls (Figure 10). The actual waveguide aperture in both cases corresponds to the WG20 waveguide and the dimensions are 10.67×4.32 mm^2^ while the length of both waveguides is 205 mm. The applied holey glide-symmetric structure follows the topology from Figure 5d and is analyzed in Figure 8d, i.e., the glide-symmetric structure is made of holes that are 7 mm in diameter and 3 mm in depth, and the periodicity of the holes is 11 mm. The actual manufactured waveguides are shown in Figure 10c,d. The structure was produced using the CNC milling machine with production tolerances around 0.01 mm (the used CNC machine is the three-axis milling machine INGPOS, Laboratory of Machine Tools, Faculty of Mechanical Engineering and Naval Architecture, University of Zagreb). The prototype was made from C45 steel by which we have ensured good flatness of the produced waveguide parts (although the conductivity of steel is smaller comparing to, e.g., aluminum).

The bandgap cut-off frequencies for different sizes of the gap are the ones given in Figure 4, and these were the basis for choosing the parameters of the developed prototype. From a practical perspective we need to cover the whole waveguide band of operation (18.0–26.5 GHz), and as seen from Figure 4, for small gap sizes (below 0.1 mm) the stop-band meets this criterion. Since the actual dimensions of the undesired gap in reality are not known, we used gap sizes of 0.05–0.2 mm in the measurements. These values should give us a good indication whether the proposed holey glide-symmetry technology can solve the abovementioned manufacturing problems with the practical cost being a slightly larger structure in some cases.

**Figure 11:**
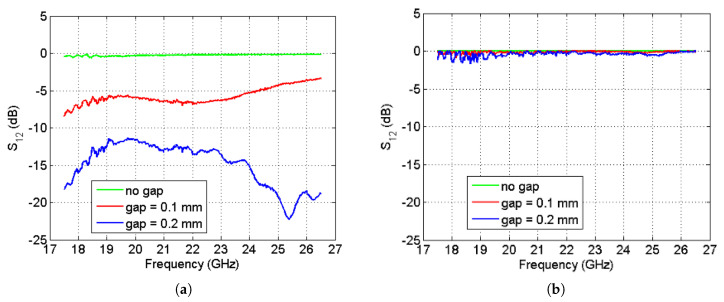
Measured $S_{21}$ parameter of the realized waveguides with different gap sizes: (**a**) ordinary waveguide; (**b**) waveguide realized using the holey glide-symmetric technology.

The S-parameters of both waveguides (classical and the one with a holey glide-symmetric structure) were measured and the magnitudes of $S_{21}$ parameters are shown in Figure 11. The results of the classical waveguide show that even a small gap (0.1 mm, which corresponds to the thickness of one 80 g/m^2^ paper sheet) causes a significant leakage of energy through the lateral walls of the waveguide. This is not the case for the glide-symmetric version of the waveguide, which efficiently eliminates the leakage problem. Furthermore, the actual experiment revealed that when using the proposed glide-symmetry technology, the required mechanical tolerances can be relaxed and the required number of screws needed to fix the waveguide can be significantly reduced.

## 4. Concept of Effective Hole Depth

When making components using propagation properties of glide-symmetric structures (e.g., lenses in PPW technology), the properties that should be considered first are the obtainable values of the effective refraction index, as well as low-dispersive and isotropic features. Once we select the basic parameters of the glide-symmetric PPW, the obtainable range of refractive indexes is determined by varying the depth of the holes. Note that for larger depths the value of the refractive index will not change due to evanescent field distribution in holes with subwavelength radius, and thus the bottom of the holes is “not visible” for the EM field.

Using a simple drilling manufacturing process for making holey glide-symmetric technology makes this technology cost-effective, in particular in the case when the fast development of proof-of-concept devices is needed. However, this means that the actual conical shape of the hole ending (due to the conical shape of the drill bit) must be taken into account in the design procedure, which is particularly important for lens realizations where the propagation properties of the EM wave in the parallel plate region depend on the hole depth [5,6,7]. In other words, there is a need for an analysis tool that can efficiently evaluate the effective depth of a circular hole with a conical ending. This is particularly important when analyzing electrically large holey glide-symmetric structures with many elements (i.e., holes), such as lenses and leaky-wave antennas.

**Figure 12:**
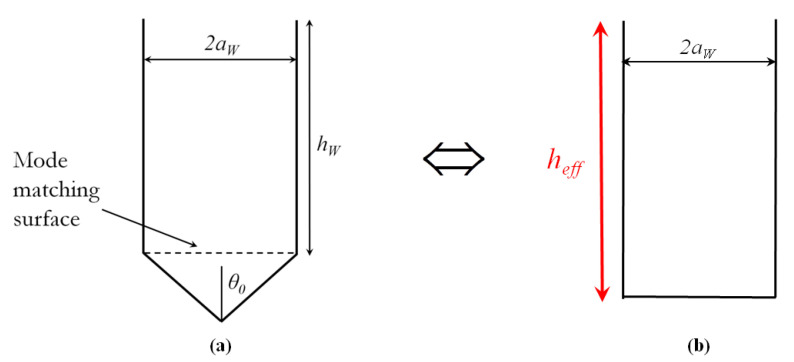
(**a**) Sketch of a hole obtained using the standard drilling procedure. Geometrically, the hole is a combination of a circular and a conical waveguide; (**b**) sketch of the equivalent hole with a straight ending and length $h_{eff}$.

The electromagnetic analysis of this problem starts by considering a combination of a circular and conical waveguide (as shown in Figure 12). Two regions are modeled using the appropriate waveguide modes with unknown amplitudes and these are then connected by applying the mode-matching technique from which the mode amplitudes can be obtained. The plane in which the mode-matching is applied is the planar boundary shown in Figure 12, and the details of the complete procedure are given in the Appendix A.

To determine the effective depth of the conical hole, we defined that the conical hole and the equivalent straight ending hole with depth $h_{eff}$ have the same reflection coefficients when excited with the considered cylindrical waveguide mode (as illustrated in Figure 12). The $h_{eff}$ is then calculated using:

(3) $$|\Gamma e^{+2j\beta_{w}h_{eff}}|=|\Gamma e^{+2\alpha_{w}h_{eff}}|=1,$$

where Γ is the reflection coefficient of the considered cylindrical evanescent waveguide mode determined through the mode-matching procedure and $\beta_{w}=-j\alpha_{w}$ is its propagation constant.

**Figure 13:**
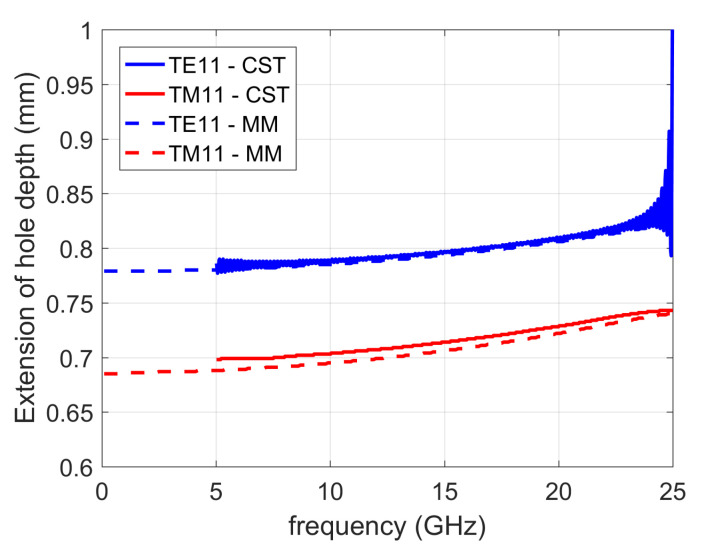
Dependency of the calculated effective extension of the hole depth on the $TE_{11}$ and $TM_{11}$ excitation modes of the cylindrical waveguide section. Solid line—CST Microwave Studio, dashed line—mode matching approach.

Figure 13 illustrates the method by showing the effective extension of the hole depth due to the conical bottom. We considered a conical shape defined with $\theta_{0}$ = 60° (a typical conical shape related to commercial drilling tools) and a hole diameter of 2*r* = 7 mm (the same as in the previous scenarios). The results are shown for both $TE_{11}$ and $TM_{11}$ waveguide mode excitations and were verified by comparison using CST Microwave Studio (for both cases, the mode-matching method and CST MS, the effective extension of the hole depth was calculated using (3)). Although the $TE_{11}$ is the dominant mode, in [33] it was shown that for lower frequencies most of the power traveling along the PPW is coupled to the “superior” TM mode(s), i.e., to the $TM_{11}$ mode in our case. Therefore, in the calculation of the effective depth of holes with conical ending we will consider the $TM_{11}$ mode. When it comes to the mode-matching formulation, that means that the $TM_{11}$ mode excites the structure and the reflection coefficient of the $TM_{11}$ mode is used in determining the effective depths, but other modes are present as well (the reflection coefficients of other modes are much smaller comparing to the one of $TM_{11}$ mode). In total we have considered 4 cylindrical and 12 spherical modes.

**Figure 14:**
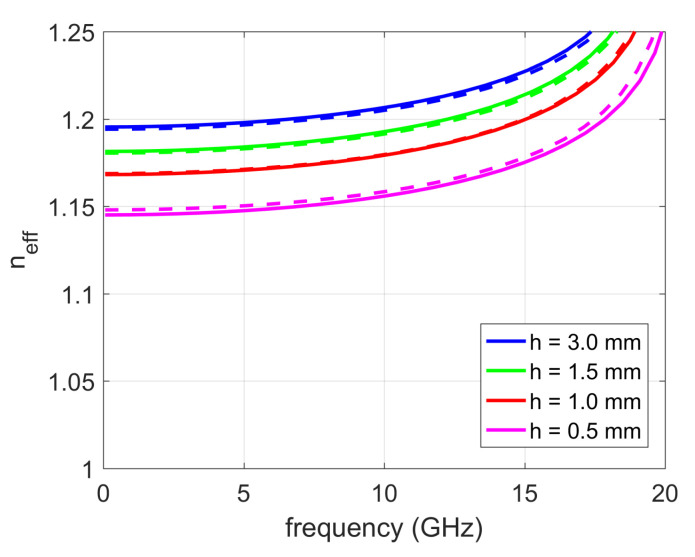
Obtained index of refraction as a function of the hole depth for holes with straight and conical endings (2D glide symmetry case). Straight line—holes with conical ending; dashed line—holes with straight ending (the hole is longer for an effective depth $h_{eff}$ equal to 0.7 mm in the considered case).

The concept of effective hole depth was verified on the 2D glide-symmetric PPW ($P^{2D}=11$ mm, 2*r* = 7 mm, *g* = 0.2 mm). As shown in Figure 14, the effective refraction index was calculated for holes with different depths, all of which have a conical ending with $\theta_{0}$ = 60°. For comparison, the refractive index of holes with straight endings and a larger depth for the $h_{eff}$ value is also shown ($h_{eff}=0.7$ mm in our case). The agreement between the obtained effective refractive indexes is very good.

## 5. Conclusions

Periodic structures with higher symmetry have shown a large potential for implementation in novel electromagnetic devices due to their possibility to tailor propagation properties of EM waves. In this paper we discussed some design features of waveguide components containing holey glide-symmetric periodic structures. In particular, we compared periodic structures with a glide symmetry in one and two dimensions. It was shown that it is much simpler to implement a 1D glide-symmetric holey structure, or a diagonally directed 2D structure, into the gap-waveguide-based components, and that in most applications it is enough to have one row of periodic holes. However, in practical realizations it is not enough to consider the stop-band properties of the holey periodic structure only, since one should also avoid Bragg frequencies due to the presence of large reflections around those frequencies. The design of a gap waveguide based on these ideas was practically realized and measured, confirming the simulated results. Finally, we have discussed a design approach of holey structures with circular-cylindrical conical endings (the case of simple production using a standard drilling manufacturing procedure), based on the effective hole depth concept, which can serve as a valuable tool in the analysis of large holey glide-symmetric structures such as lenses and leaky-wave antennas.

## Appendix A

In this Appendix we will present the analysis procedure for determining the effective hole depth of the cylindrical hole with the conical shape of the hole bottom. Geometrically, such a hole consists of a circular and a conical waveguide sections (see Figure 12). Electromagnetically, each waveguide section can be modeled with inherent waveguide modes, and their amplitudes are determined by applying the mode-matching technique. In more detail, the conical section can be modeled by the following modes:

(A1) $$E_{r}=-j\frac{\eta_{0}}{k_{0}}(\frac{\partial^{2}}{\partial r^{2}}+k_{0}^{2})A_{r}$$

(A2) $$E_{\theta }=-j\frac{\eta_{0}}{k_{0}}\frac{1}{r}\frac{\partial^{2}A_{r}}{\partial r\partial \theta }-\frac{1}{r\sin\theta }\frac{\partial F_{r}}{\partial \phi }$$

(A3) $$E_{\phi }=-j\frac{\eta_{0}}{k_{0}}\frac{1}{r\sin\theta }\frac{\partial^{2}A_{r}}{\partial r\partial \phi }+\frac{1}{r}\frac{\partial F_{r}}{\partial \theta }$$

(A4) $$H_{r}=-j\frac{1}{\eta_{0}k_{0}}(\frac{\partial^{2}}{\partial r^{2}}+k_{0}^{2})F_{r}$$

(A5) $$H_{\theta }=-j\frac{1}{\eta_{0}k_{0}}\frac{1}{r}\frac{\partial^{2}F_{r}}{\partial r\partial \theta }+\frac{1}{r\sin\theta }\frac{\partial A_{r}}{\partial \phi }$$

(A6) $$H_{\phi }=-j\frac{1}{\eta_{0}k_{0}}\frac{1}{r\sin\theta }\frac{\partial^{2}F_{r}}{\partial r\partial \phi }-\frac{1}{r}\frac{\partial A_{r}}{\partial \theta }$$

Here $A_{r}$ and $F_{r}$ are the radial components of the magnetic and electric potential, which are related to the considered mode in the form:

(A7) $$A_{r}\left(r,\theta \phi \right)=\alpha_{n,m}\cdot {\hat{J}}_{m}\left(k_{0}r\right)P_{\nu_{n}}^{m}\left(\cos\theta \right)e^{-jm\phi }$$

(A8) $$F_{r}\left(r,\theta \phi \right)=\beta_{n,m}\cdot {\hat{J}}_{m}\left(k_{0}r\right)P_{\nu_{n}^{\prime }}^{m}\left(\cos\theta \right)e^{-jm\phi },$$

where ${\hat{J}}_{n}$ and $P_{\nu }^{m}$ denote Schelkunoff’s spherical Bessel functions and the associated Legendre functions, respectively [34]. As can be seen from Equations (A7)and (A8), the potentials and consequently the EM field distribution depend on the indices $\nu_{n}$ and $\nu_{n}^{\prime }$. These two indices of the associated Legendre functions are in general non-integer numbers obtained by imposing the appropriate boundary conditions on the cone metallic surface, i.e., the tangential electric field vanishes when $\theta =\theta_{0}$. In other words, the indices $\nu_{n}$ and $\nu_{n}^{\prime }$ are calculated from the following equations:

(A9) $$\begin{array}{cc} P_{\nu_{n}}\left(\cos\theta \right)=0 & \mathrm{in}\mathrm{TM}\mathrm{case} \end{array}$$

(A10) $$\begin{array}{cc} \frac{\partial P_{\nu_{n}^{\prime }}\left(\cos\theta \right)}{\partial \theta }=0 & \mathrm{in}\mathrm{TE}\mathrm{case} \end{array}$$

The roots of Equations (A9) and (A10) are numerically determined by implementing the recursive equations for evaluating the associated Legendre functions together with Newton’s algorithm for root finding, and the resulting combination enables high computation precision.

The circular waveguide is modeled with “classical” waveguide modes determined by the following form of the z-component of the magnetic and electric vector potentials [34]:

(A11) $$A_{z}\left(\rho ,\phi ,z\right)=\xi_{m,l}\cdot J_{m}\left(k_{\rho }\rho \right)e^{-jm\phi }e^{\pm jk_{z}z}$$

(A12) $$F_{z}\left(\rho ,\phi ,z\right)=\zeta_{m,l}\cdot J_{m}\left(k_{\rho }\rho \right)e^{-jm\phi }e^{\pm jk_{z}z}.$$

The tangential EM fields are determined as:

(A13) $$E_{\rho }=-j\frac{\eta_{0}}{k_{0}}\frac{\partial^{2}A_{z}}{\partial \rho \partial z}-\frac{1}{\rho }\frac{\partial F_{z}}{\partial \phi }$$

(A14) $$E_{\phi }=-j\frac{\eta_{0}}{k_{0}}\frac{1}{\rho }\frac{\partial^{2}A_{z}}{\partial \phi \partial z}+\frac{\partial F_{z}}{\partial \rho }$$

(A15) $$H_{\rho }=-j\frac{1}{\eta_{0}k_{0}}\frac{\partial^{2}F_{z}}{\partial \rho \partial z}+\frac{1}{\rho }\frac{\partial A_{z}}{\partial \phi }$$

(A16) $$H_{\phi }=-j\frac{1}{\eta_{0}k_{0}}\frac{1}{\rho }\frac{\partial^{2}F_{z}}{\partial \phi \partial z}-\frac{\partial A_{z}}{\partial \rho }.$$

Here $k_{z}=\sqrt{k_{0}^{2}-k_{\rho }^{2}}$ and $J_{m}$ denote the Bessel functions of the first kind. The value of $k_{\rho }$ depends on the considered waveguide mode and is determined by imposing the boundary condition that the tangential electric field vanishes at the waveguide walls, i.e., $J_{m}\left(k_{\rho }a\right)=0$ for the TM modes and $J_{m}^{\prime }\left(k_{\rho }a\right)=0$ for the TE modes. The mode matching is performed over the planar boundary of the cylindrical waveguide (see Figure 12). As testing functions we have selected the *H*-field distribution of spherical weaveguide modes and *E*-field distribution of the cylindrical waveguide modes (details about implementation of the mode-matching procedure can be found in [35]).

The effective depth of the hole $h_{eff}$ is determined as follows. Once we determine by the mode-matching procedure the reflection coefficient Γ of the considered cylindrical evanescent waveguide mode (with propagating constant $\beta_{w}=-j\alpha_{w}$), the effective depth of the hole $h_{eff}$ is calculated using the following equation:

(A17) $$|\Gamma e^{+2j\beta_{w}h_{eff}}|=|\Gamma e^{+2\alpha_{w}h_{eff}}|=1$$

Note that this equation actually states that the reflection coefficients of the considered hole with the conical ending and of the equivalent hole with the straight ending (of depth $h_{eff}$) are equal. This concept is also illustrated in Figure 12.
